# Editorial: Nutrition and Behavior as Determinants of Host-Associated Microbiomes

**DOI:** 10.3389/fmicb.2021.835394

**Published:** 2022-02-21

**Authors:** Yvonne Vallès, M. Pilar Francino

**Affiliations:** ^1^The University of the West Indies-Cave Hill Campus, Cave Hill, Barbados; ^2^Fundación para el Fomento de la Investigación Sanitaria y Biomédica de la Comunitat Valenciana (FISABIO), Valencia, Spain; ^3^CIBER en Epidemiología y Salud Pública, Madrid, Spain

**Keywords:** animal, microbiome, modulation, nutrition, behavior

Symbiotic relationships are dynamic in that adjustments are continuously made by the sets of organisms involved in the association. When considering microbiomes and their hosts, factors such as nutrition, behavior and a changing environment will affect both the host and its microbiome's composition, dynamics, and function. As our understanding of the role of microbiomes in the host's health grows, so does the thirst for knowledge on mechanisms of microbiome modulation. Here we present a collection of works that range from exploring natural microbiome variability under different geographical regions, to evaluating the effects of dietary and psychosocial factors on the human microbiome, to developing potential nutritional approaches to improve human and animal health and to increase efficiency and productivity in various animal systems.

As an example of natural microbiome variability, Liu et al. explore microbiome composition in three populations of yaks that inhabit three different regions of the environmentally heterogeneous Qinghai-Tibetan plateau. The Qinghai-Tibetan plateau is the largest and highest plateau in the world enclosing regions that vary vastly in altitude [from 500 m above sea level (a.s.l) at the foot of the Himalaya to 8,848 m a.s.l. at mount Everest], annual precipitation (from areas with 300 mm to others with 3,000 mm) and temperature, among other environmental factors (Favre et al., 2014). Yet, it is the home of 12 yak breeds, an extremely hardy indigenous ruminant found across the plateau (Liu et al.). Fascinatingly, Liu et al. demonstrate that the microbiome's diversity and composition will reflect the geographical region the yak population inhabits. Importantly, the soil of the Qinghai-Tibetan plateau is low in selenium (Se), so that Se deficiency is widespread in the yak and sheep that graze in this extreme environment. Cui et al. show that Se supplementation to the forage-based feed of Tibetan sheep improves fermentation and rumen microbial community structure, increasing livestock health and productivity.

In view of the indirect and unwanted connection of ruminants to climate change, Bharanidharan et al. explore the associations among cattle feeding systems (separate feeding vs. total mixed ration), breeds, rumen microbiota composition and CH_4_ emissions. Interestingly, breeds independently of the feeding system have differential rumen microbial communities, with Hanwoo steers being higher CH_4_ emitters than Holstein steers, but the use of a separate feeding system is more effective at reducing CH_4_ emissions regardless of the cattle breed.

The gut microbiota also has an important effect on the nutritional physiology of pigs. Petry et al. and Long and Venema explore the possibility of modulating the swine gut microbiota by feeding the pigs with a corn-based feed supplemented with xylanase and a chemically pre-treated rapeseed meal, respectively. These studies come as a necessity, as the diet used in the swine industry presents a high proportion of dietary fiber, which the pig's intestine has no capacity to metabolize, decreasing the productivity of the swine industry. In both cases, the supplementation of corn-based feed with xylanase and the chemical pre-treatment of rapeseed increase microbial community diversity and fiber degradability potential.

Focussing on a different food production industry, Huang et al. examine the effect of cassava starch-based feed on the microbiome composition and intestinal health of a carnivorous fish, the largemouth bass. Starch is an inexpensive feed ingredient, but carnivorous fishes are not well-adapted to its use. Interestingly, a significant decrease of beneficial bacteria abundance is observed in fish fed with a high-starch diet, with subsequent development of enteritis. To modulate the microbiome and prevent the development of enteritis, the authors propose the use of probiotics, reintroducing the lost beneficial bacteria and increasing productivity.

The influence of diet on the microbiome is not only of importance in animals raised for food but also in animal pets. The impact of modern lifestyles, characterized by higher energy intake packaged in smaller food bites with an increase in sedentarism, affects our pets, as a reflection of human behavior and nutrition. Therefore, current trends of increase in disorders such as diabetes and obesity are not unique to humans but extend to our pets as well. In their study, Li and Pan, examine the effect of a high protein low carbohydrate (HLPC) diet recommended for weight management vs. a control diet (CON: higher carbohydrate percentage with lower protein percentage) on the gut microbiome of lean and obese cats. Interestingly, the different diets do not influence the lean cat's microbiota, while significant differences are observed between the obese cats fed with HPLC and CON diets, where the HPLC-fed obese cat's microbiome has a greater abundance of short-chain fatty acid producers that could eventually be used in weight management therapies.

Overall, these various studies in animal systems illustrate the vast potential of microbiome modulation as a strategy to improve health in both pets and farm animals, as well as productivity in animal-based food industries.

Finally, we report several studies on the human's gut microbiome that (1) validate once again the fact that there is an inherent individual variability in microbiota functionality, as shown by Riva et al. regarding individual ability to metabolize rutin, a bioactive polyphenolic compound with antioxidant, antiaging, and anti-neurodegenerative properties; (2) demonstrate the possibility of positively modulating the microbiome by adding tannin wood extracts to food and in particular quebracho and tara, as Molino et al. show that their addition is significantly associated to an increased presence of *Akkermansia* and beneficial short-chain fatty acid producers; and (3) indicate that the disruption of an equilibrated microbiome is affected by more than just physical cues, with psychological and behavioral cues as well-influencing microbiome development. In their study, Rodriguez et al. show that the antenatal psychological status of the mother can disrupt the infant's gut microbiome through the transmission of a suboptimal maternal microbial community, a negative effect on breast milk immune properties, and an adverse impact on the duration of breastfeeding.

## Author Contributions

All authors listed have made a substantial, direct, and intellectual contribution to the work and approved it for publication.

## Conflict of Interest

The authors declare that the research was conducted in the absence of any commercial or financial relationships that could be construed as a potential conflict of interest.

## Publisher's Note

All claims expressed in this article are solely those of the author and do not necessarily represent those of their affiliated organizations, or those of the publisher, the editors and the reviewers. Any product that may be evaluated in this article, or claim that may be made by its manufacturer, is not guaranteed or endorsed by the publisher.
